# Optical photothermal infrared spectroscopy with simultaneously acquired Raman spectroscopy for two-dimensional microplastic identification

**DOI:** 10.1038/s41598-022-23318-2

**Published:** 2022-11-05

**Authors:** Julia Sophie Böke, Jürgen Popp, Christoph Krafft

**Affiliations:** 1 Leibniz Institute of Photonic Technology, Albert-Einstein-Str. 9, 07745 Jena, Germany; 2 Institute of Physical Chemistry and Abbe Center of Photonics, Friedrich-Schiller University Jena, Helmholtzweg 4, 07743 Jena, Germany

**Keywords:** Environmental sciences, Optics and photonics

## Abstract

In recent years, vibrational spectroscopic techniques based on Fourier transform infrared (FTIR) or Raman microspectroscopy have been suggested to fulfill the unmet need for microplastic particle detection and identification. Inter-system comparison of spectra from reference polymers enables assessing the reproducibility between instruments and advantages of emerging quantum cascade laser-based optical photothermal infrared (O-PTIR) spectroscopy. In our work, IR and Raman spectra of nine plastics, namely polyethylene, polypropylene, polyvinyl chloride, polyethylene terephthalate, polycarbonate, polystyrene, silicone, polylactide acid and polymethylmethacrylate were simultaneously acquired using an O-PTIR microscope in non-contact, reflection mode. Comprehensive band assignments were presented. We determined the agreement of O-PTIR with standalone attenuated total reflection FTIR and Raman spectrometers based on the hit quality index (HQI) and introduced a two-dimensional identification (2D-HQI) approach using both Raman- and IR-HQIs. Finally, microplastic particles were prepared as test samples from known materials by wet grinding, O-PTIR data were collected and subjected to the 2D-HQI identification approach. We concluded that this framework offers improved material identification of microplastic particles in environmental, nutritious and biological matrices.

## Introduction

The necessary step towards reproducible microplastic (MP) identification and quantification lies in comparing studies of different laboratories and instruments. In recent years, research has evolved from relying on visual inspection, which identified only 1.4% of the suspected particles correctly as synthetic polymers^1^, over manual analysis based on vibrational spectroscopy^2^, to automated measurement approaches^3,4^. Based on vibrational spectroscopy, techniques such as Raman and Fourier transform infrared (FTIR) spectroscopy determine and differentiate between different polymer types^5–10^. Nevertheless, relying on FTIR spectroscopy only can lead to an underestimation of 32% compared to Raman spectroscopy^11^ which is most likely due to limited spatial resolution of traditional FTIR. With advances in analytical techniques and a growing interest in MP research, the need for reliable detection methods and the need for standardization have been rising^12–16^. Modern analysis methods such as machine learning were applied to spectral data of MPs^17^ and demonstrated to minimize identification errors and improve the accuracy of the data analysis^18,19^. The international organization of standards summarizes the state of knowledge and methodology of MP detection and methods for analysis^20^.

IR and Raman spectroscopy are complementary methods to some extent. IR spectroscopy targets the vibrations of molecular bonds upon a change in dipole moment. IR-active vibrations can be excited by absorption of mid-IR radiation. For example, C–H, C–O and C=O groups in plastic polymers change their permanent dipole moment during vibrations, making them suitable for IR detection. Although some molecular groups do not have a dipole moment in the equilibrium state, a dipole can be induced making them IR-active, which is, for example, the case for the antisymmetric stretch vibration of CO_2_. FTIR spectra can be acquired in transmission and reflection mode, both allowing non-contact operation. The attenuated total reflection (ATR) mode requires close contact with the internal reflection element due to the low propagation range of the evanescent field. For IR wavelengths of prominent C=O bands in the fingerprint range near 1700 cm^−1^ corresponding to 5.9 µm wavelength, and a numerical aperture (NA) of typical all-reflective IR objective lenses ranging from 0.4 to 0.8, the resolution limit ranges from 7.4 to 3.7 µm according to the Abbe's equation of diffraction limit for microscopes. This is one order of magnitude coarser than Raman spectroscopy due to excitation wavelengths in the visible spectral range. With IR spectroscopy, microparticles above 20 µm are detectable in reflectance or transmittance mode on compatible substrates^6,21^. FTIR microscopes with a focal plane array (FPA) detector enable the collection of images of extended regions of interest with diffracted-limited spatial resolution^22,23^. With the advent of mid-IR quantum cascade lasers (QCLs), new IR instruments were developed which utilize high intensity, high brilliance, narrow bandwidth and tunability of QCLs. These features enable widefield microscopic imaging with a large (480 × 480 pixels), uncooled microbolometer array, which is much faster than FTIR microscopic imaging. This method allowed the measurement of MPs in an area of 144 mm^2^ in 36 min with a pixel resolution of 4.2 µm^24^. Another approach couples mid-IR QCLs with a thermoelectrically-cooled single-channel detector for rapid chemical imaging with a fully automated workflow. This approach has been compared with FPA-FTIR imaging for microplastic detection^15^. As these FTIR and emerging QCL-based systems detect directly the transmitted or reflected IR radiation of the sample, they can be called direct IR approaches that suffer—besides the above-described resolution in the micrometer range – from Mie scattering and anomalous dispersion effects, in particular for sample sizes near the IR wavelengths 12 to 5.5 µm corresponding to the most informative spectral fingerprint range 800 to 1800 cm^−1^.

Raman spectroscopy provides information about the change of polarizability of the chemical bond upon molecular vibrations. In particular, vibrations of C–C-, C=C-bonds, and aromatic rings in polymers introduce polarizability changes. Thus, it is suitable for label-free identification of MP particles^25^. While Raman spectroscopy has many advantages, such as submicron spatial resolution, it is highly susceptible to fluorescence interferences that can be particularly prevalent in environmental samples and might be attributed to degradation effects or adsorbed contaminations. Fluorescence emission can superimpose the usually weaker Raman scattered signal with a broad background and thus prevent or complicate reliable identification of the particle^26^. Additionally, the Raman spectra of colored and pigmented MP samples might be distorted by the fluorescent emission or resonant enhanced Raman signals of the dye dominating the fingerprint signature of the plastic particle. Raman systems can assess particles ranging from 0.5 µm to a few mm in size. Laser excitation usually in the visible wavelength between 400 and 800 nm range enables compatibility with suspended samples and aqueous solutions due to the relatively weak Raman scattering of water. Although single Raman spectra of plastic can be acquired within a few milliseconds, acquiring large area Raman images of millions of points in the serial scanning mode is experimentally slow. Therefore, common strategies determine the particle position and collect single Raman spectra from each particle recently shown for single cells^27^. Another strategy is coherent Raman scattering with dwell times for single bands in the microsecond range, which has recently been shown for plastic materials^28^.

Käppler et al. compared the suitability of Raman and FTIR spectroscopy for MP identification^11^. They concluded that a dual analysis is unlikely to lead the routine analysis due to its time and cost, even though it would result in the highest certainty. In this paper, we demonstrate a systematic comparison of IR (FTIR and O-PTIR) and Raman spectra (collected via simultaneous O-PTIR + Raman and standalone Raman) and their reliability as the primary source of identification of MP. In contrast to the above introduced direct IR spectroscopy, O-PTIR spectroscopy uses an indirect detection scheme: (i) a pulsed pump mid-IR QCL induces photothermal effects in the sample, (ii) absorption of radiation is followed by small local heating resulting in thermal expansion and a change in refractive index, (iii) a visible probe laser is focused on the sample which detects these photothermal IR effects as well as acting as simultaneous Raman excitation laser which enables acquisition of both IR and Raman spectra from the same measurement location at the same time with the same spatial resolution. For solid samples, the O-PTIR technique is often operated in reflection mode, which confers many practical advantages with the spectra comparable to thin-film FTIR spectra acquired in transmission mode. First, we analyzed the spectra of eight common polymers, polyethylene (PE), polypropylene (PP), polyvinyl chloride (PVC), polyethylene terephthalate (PET), polycarbonate (PC), polystyrene (PS), silicone, polymethylmethacrylate (PMMA), and one biopolymer polylactic acid (PLA) with standalone Raman and FTIR instruments, and presented comprehensive band assignments. We compared the spectra from the O-PTIR measurements with standalone ATR-FTIR and also compared the simultaneously (with O-PTIR) acquired Raman with standalone Raman spectrometers based on the hit quality index (HQI). Second, we introduce a two-dimensional (2D) HQI approach that uses both HQIs of IR and Raman spectra for a more robust and accurate MP identification. Finally, MP particles were prepared as test samples from known material by wet grinding, O-PTIR data were collected and subjected to the 2D-HQI identification approach. As the O-PTIR instrument can acquire simultaneous IR and Raman spectra, this offers a clear advantage for identifying MP particles.

## Results

### Comparison between FTIR and O-PTIR

**Figure 1:**
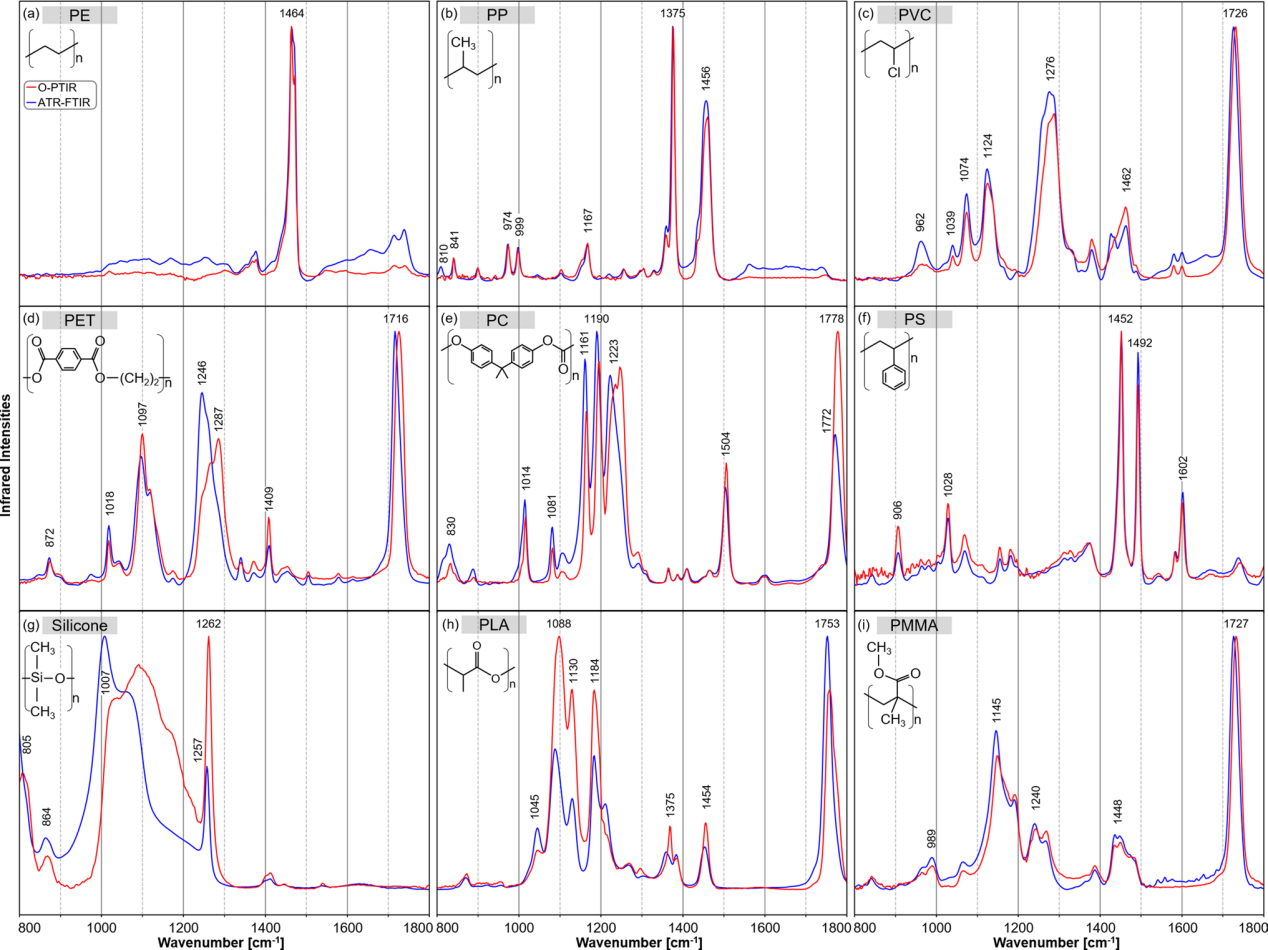
Normalized IR spectra from 800 to 1800 cm^−1^ of different plastics: polyethylene (PE) (**a**), polypropylene (PP) (**b**), polyvinyl chloride (PVC) (**c**), polyethylene terephthalate (PET) (**d**), polycarbonate (PC) (**e**), polystyrene (PS) (**f**), silicone (**g**), polylactide acid (PLA) (**h**) and polymethylmethacrylate (PMMA) (**i**) collected with the standalone ATR-FTIR system (blue) and the O-PTIR instrument collected in reflection mode (red).

In this section, we compare the characteristics of FTIR with O-PTIR spectra. Although O-PTIR spectra were collected in reflection mode, they correspond to FTIR spectra of thin films in transmission mode. For comparison, an ATR correction was applied to the spectra of the standalone FTIR instrument collected in ATR mode. This accounts for the dependence of optical path length d on the wavelength λ. Details can be found in the 'Materials and Methods' section. IR spectra in Fig. 1 are shown for the spectral range from 800 to 1800 cm^−1^. They were acquired with the standalone ATR-FTIR system (blue) and the O-PTIR instrument (red).

PE had its main IR band near 1464 cm^−1^ (Fig. 1a), resulting from the methylene deformation vibrations. The C-H groups were the only bonds with a permanent dipole initiating vibrations. Overall, the FTIR spectrum was in good agreement with the O-PTIR spectrum, though minor differences in the background and relative band intensities were observed.

Key IR bands of PP are related to methyl deformation vibrations near 1456 and 1375 cm^−1^ (Fig. 1b). Weaker IR bands were detected in the lower wavenumber region between 800 and 1200 cm^−1^ that were assigned to C–C (808, 974 cm^−1^), and C–H (841, 999, 1167 cm^−1^). FTIR and O-PTIR spectra agreed very well.

Figure 1c shows the IR spectra of PVC. Methylene deformation vibrations of CH_2_ occurred at 1462 cm^−1^, similar to the IR spectrum of PE. The bands at 1074, 1276 and 1124 cm^−1^ correspond to C–O bands. The one near 1726 cm^−1^ is related to C=O modes, which were unexpected in PVC as the main structure lacks such functional groups. However, they are common bands in PVC containing plasticizers^29^ that can contribute up to 80% by weight in flexible tubes. The heavy chlorine atoms led to C–Cl vibrations usually observed in the wavenumber range below 800 cm^−1^. Typical HC–Cl bands were expected near 1250 cm^−1^ but overlapped with C–O bands of the plasticizers.

The IR spectra of PET in Fig. 1d show variations in the relative intensities of C–O ester bands between 1050 and 1300 cm^−1^ with maxima at 1246 and 1287 cm^−1^. The respective band for the C=O ester groups was found at 1726 cm^−1^ in the O-PTIR spectrum, while the band was shifted to 1716 cm^−1^ in the FTIR spectrum. Differences might be due to surface orientation effects^30^ as the spectra could not be obtained from the same position. The C–C band of the phenyl ring at 1409 cm^−1^ occurred in both spectra at the same position. Although structurally different from PET, O-PTIR spectra of PVC and PET shared some similarities, e.g. bands near 1120 and 1280 cm^−1^, which resulted from the plasticization of PVC.

In Fig. 1e, the IR bands of carbonyl bonds in PC near 1780 cm^−1^ were found at higher wavenumbers relative to carbonyl groups in PET and PMMA. The bands between 1161 and 1240 cm^−1^ originated from the ester groups of the molecules. Their shift towards lower wavenumbers compared to PET resulted from the more ether-like character of the C-O bond. A unique spectral feature of PC was a band at 1504 cm^−1^, assigned to an aromatic ring vibration. The FTIR and O-PTIR spectra had a similar signature but varied band intensities and maxima.

**Figure 2:**
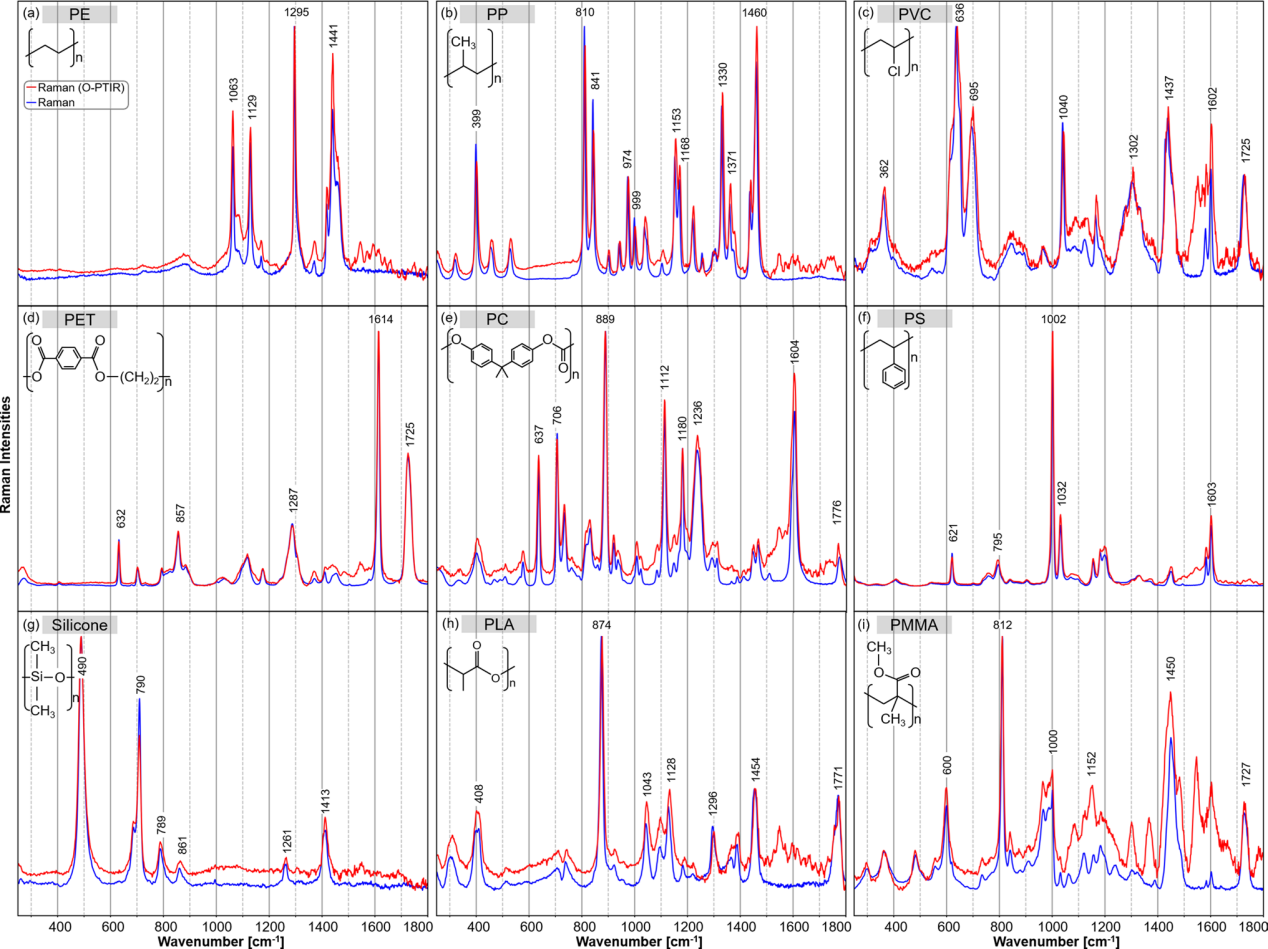
Normalized Raman spectra from 250 to 1800 cm^−1^ of different plastics: polyethylene (PE) (**a**), polypropylene (PP) (**b**), polyvinyl chloride (PVC) (**c**), polyethylene terephthalate (PET) (**d**), polycarbonate (PC) (**e**), polystyrene (PS) (**f**), silicone (**g**), polylactide acid (PLA) (h) and polymethylmethacrylate (PMMA) (**i**) collected with the standalone Raman system (blue) and the simultaneous Raman option of the O-PTIR instrument (red).

Figure 1f shows the IR spectra of PS. Prominent IR bands at 1602 and 1492 cm^−1^ are related to aromatic ring vibrations and at 1452 cm^−1^ to methylene vibrations. The bands at 1492 and 1452 cm^−1^ were dipole-active only and thus, had no counterparts in the Raman spectrum (see Fig. 2f). The band at 1028 cm^−1^ corresponded to the in-plane CH bending of the aromatic ring^31^. The spectra acquired with the FTIR and O-PTIR systems are in good agreement.

The IR bands of silicone were dominated by the Si–O stretching vibration between 1000 and 1200 cm^−1^ with a maximum near 1007 cm^−1^ and the symmetric Si-CH_3_ bending vibration at 1260 cm^−1^ (Fig. 1g). Another band of Si-CH_3_ in the O-PTIR spectrum near 805 cm^−1^ was shifted towards 785 cm^−1^ for the FTIR instrument, and just the slope was evident. The band corresponds to the symmetric out-of-plane bending of Si-CH_3,_ whereas the band of the antisymmetric bending vibration was found at 864 cm^−1^^32^. The higher mass of the silicone atoms induced a shift of the main bands to lower wavenumbers compared to hydrocarbon polymers. The FTIR and the O-PTIR spectra showed some mismatch in band intensities and maxima, which probably arises from probing different positions of the bulk polymer after a sample transfer between instruments.

The C–O bond of the ester vibrations of PLA and their coupling effects in the polymer chain were associated with maxima at 1088, 1130 and 1184 cm^−1^ in Fig. 1h. The bands located at 1375 and 1454 cm^−1^ originated from the methyl group. The carbonyl group also had IR bands near 1753 cm^−1^.

The IR spectra of PMMA are presented in Fig. 1i. IR bands at 1145 and 1240 cm^−1^ were assigned to C–O vibrations in the ester group. Another band at 989 cm^−1^ was assigned to a C–O–CH_3_ vibration of the ester group. Bands of methyl vibrations occur near 1448 cm^−1^. Similar to the carbonyl bands of PET and PC, an IR band of C=O stretch vibration of the carbonyl was found near 1727 cm^−1^. PMMA has similar functional groups to PLA.

Overall, both the FTIR and O-PTIR instruments conserved the position of the IR bands well, and the spectra are in good agreement. The quantitative analysis of their HQI values will confirm the resemblance of the spectra (see section “Hit quality index of the spectra”). Relative changes in intensity might be due to local variations in the chemical composition. Due to the transfer of samples between instruments, different positions have been probed.

### Comparison between Raman and Raman option of O-PTIR

In comparison to IR spectroscopy, Raman scattering allowed detecting an extended wavenumber range below 800 cm^−1^ and down to 250 cm^−1^. In this section, we compare the spectral characteristics of Raman measurements (blue) with the Raman scattering collected simultaneously with O-PTIR (red) in Fig. 2. Whereas the standalone Raman system performed an automatic intensity calibration, the spectra of the O-PTIR Raman option were manually calibrated to compensate for intensity variations due to different optical components such as the Cassegrain objective lens and quantum efficiency of the CCD detector, and to compare both Raman spectra properly. In particular between 1500 and 1800 cm^−1^, the O-PTIR instrument had a lower collection efficiency than the standalone Raman system, and a larger calibration factor is applied. As a result, the signal-to-noise-ratio (SNR) is similar between 250 and 1500 cm^−1^ and decreases in the Raman spectra of the O-PTIR instrument with increasing wavenumber. Details are provided in the 'Materials and Methods' section and raw Raman spectra are presented in the supplementary material.

Figure 2a shows the Raman spectra of PE. Bands due to CH_2_ deformations were found at 1295, 1418, 1441 cm^−1^, and a shoulder near 1460 cm^−1^. The latter band was evident at a similar position in the IR spectrum in Fig. 1a. Additional bands in the Raman spectra originated from the C–C bonds' good polarizability, leading to vibrations near 1063 and 1129 cm^−1^. The relative band intensities between the O-PTIR system and the standalone Raman device differed slightly and the band positions showed a good agreement.

The main Raman bands of PP were located near 399, 810, 841, 1153, 1330, 1371, and 1460 cm^−1^ (Fig. 2b). Most Raman bands were more intense than IR bands. Their intensities changed relative to the methyl-associated bands at 1371 and 1460 cm^−1^. Bands at 399, 810, 841 and 1330 cm^−1^ were assigned to various CH and CH_2_ deformation vibrations, and bands at 974 and 999 cm^−1^ to CH_3_ deformation vibrations. Bands at 1153 and 1168 cm^−1^ are related to CC stretch vibrations. The spectra of the standalone Raman and the O-PTIR instrument were in good agreement considering their relative intensities.

The prominent Raman bands of PVC are presented in Fig. 2c. The heavy molecular mass of chlorine led to additional Raman bands of C–Cl vibrations in the lower wavenumber region at 636 and 695 cm^−1^. The bands around 1302 and 1437 cm^−1^ were assigned to the methylene groups. The Raman band near 1725 cm^−1^ of the carbonyl group was consistent with the spectral contributions of plasticizers that were already observed in IR spectra. In addition, Raman bands at 1040 and 1602 cm^−1^ were typical for the common plasticizer phthalate.

In Fig. 2d, the spectra of PET show their main Raman bands at 1116 and 1287 cm^−1^ of C(O)–O vibrations in the ester group and C=O vibration of the carbonyl group at 1725 cm^−1^. The aromatic ring vibration at 1614 cm^−1^ was only present in the Raman spectrum (Fig. 2d) and not in the IR spectrum (Fig. 1d). The spectra of the standalone Raman and the O-PTIR instrument coincided well.

The Raman spectra of PC are presented in Fig. 2e. The C–O–C vibrations in ester groups observed in IR spectra have Raman counterparts at 1112, 1180 and 1236 cm^−1^. The intense Raman band at 889 cm^−1^ was assigned to an O–C(O)–C stretching vibration, which gave only a weak IR signal. Conversely, the Raman band at 1776 cm^−1^ of the C=O stretching vibration was weak but very intense in the IR spectrum in Fig. 1e. Vibrations of methyl groups showed relatively weak Raman bands near 1450 cm^−1^. The Raman band near 1604 cm^−1^ is due to the carbon–carbon vibration of the aromatic rings. Raman bands at 637 cm^−1^ (phenyl ring) and 706 cm^−1^ (CH out-of-plane bending vibration) were also typical of the aromatic moiety.

The PS Raman spectrum (Fig. 2f) was dominated by vibrational bands of the aromatic ring at 621 cm^−1^ (ring deformation mode), 795 cm^−1^ (CH out-of-plane deformation), 1002 cm^−1^ (symmetric ring breathing mode), 1032 cm^−1^ (CH in-plane deformation) and 1603 cm^−1^ (ring-skeletal stretch). Both Raman spectra were in good agreement. They show the distinctive narrow band characteristics of PS, which is recommended as a Raman standard for instrument calibration^33^. It was also the first polymer to produce a Raman spectrum^34^. Thus, it underlined the precision of both instruments and the performed intensity calibration, as the spectra overlay almost perfectly.

Main Raman bands of silicone (Fig. 2g) were located at 490 cm^−1^ (symmetric Si–O stretch vibration) of the Si–O–Si chain and 710 cm^−1^ (symmetric C–Si–C stretch vibration) and 789 cm^−1^ (antisymmetric C–Si–C stretch vibration) of the CH_3_-Si-CH_3_ moieties. Further bands at 861, 1261 and 1413 cm^−1^ were assigned to CH_3_ vibrations. Due to the larger atomic mass of Si, the Si-related bands occurred in the lower wavenumber range. Most Raman bands had smaller bandwidths than IR bands.

In Fig. 2h, the most intense band of PLA at 874 cm^−1^ was assigned to a C-COO vibration. A related C–CO vibration showed a band at 408 cm^−1^^35^. In contrast to IR spectra, C–O bond ester vibrations in PLA were weak, and the Raman bands with maxima at 1043 and 1128 cm^−1^ were associated with C–CH_3_ vibrations. The Raman bands located at 1296 and 1454 cm^−1^ originated from CH and CH_3_ deformation vibrations, respectively. The carbonyl group had Raman bands at 1771 cm^−1^.

Raman spectra of PMMA are presented in Fig. 2i. The carbonyl bonds occurred at 1727 cm^−1^. The ester vibrations of the C–O group were associated with bands between 1145 and 1240 cm^−1^. Except for two additional bands around 1152 cm^−1^ with the O-PTIR device, both Raman spectra agreed well. The PMMA has similar functional groups to PLA, which is also evident in the spectral comparison with Fig. 2h and i.

After intensity calibration, most Raman spectra acquired from the standalone Raman instrument coincide very well with the simultaneous Raman option of the O-PTIR instrument. Only minor differences in relative band intensities were observed. A quantitative comparison of spectra using the hit quality index is described in the following section.

### Hit quality index of the spectra

The hit quality index (HQI) is defined to compare the resemblance of two vectors and is established as a common measure to match an acquired spectrum X with a library spectrum Y through^36^

1 $$HQI = \frac{{\left( {X \cdot Y} \right)^{2} }}{{X^{2} \cdot Y^{2} }}.$$

The HQI value is a measure of orthogonality. If the spectra are considered vectors, an $HQI=1.0$ is associated with parallel vectors with high linear dependence. Thus, the spectra are alike. If the vectors are oriented perpendicular to each other, they are linearly independent, and the $HQI=0.0$. The spectra would have no resemblance.

**Figure 3:**
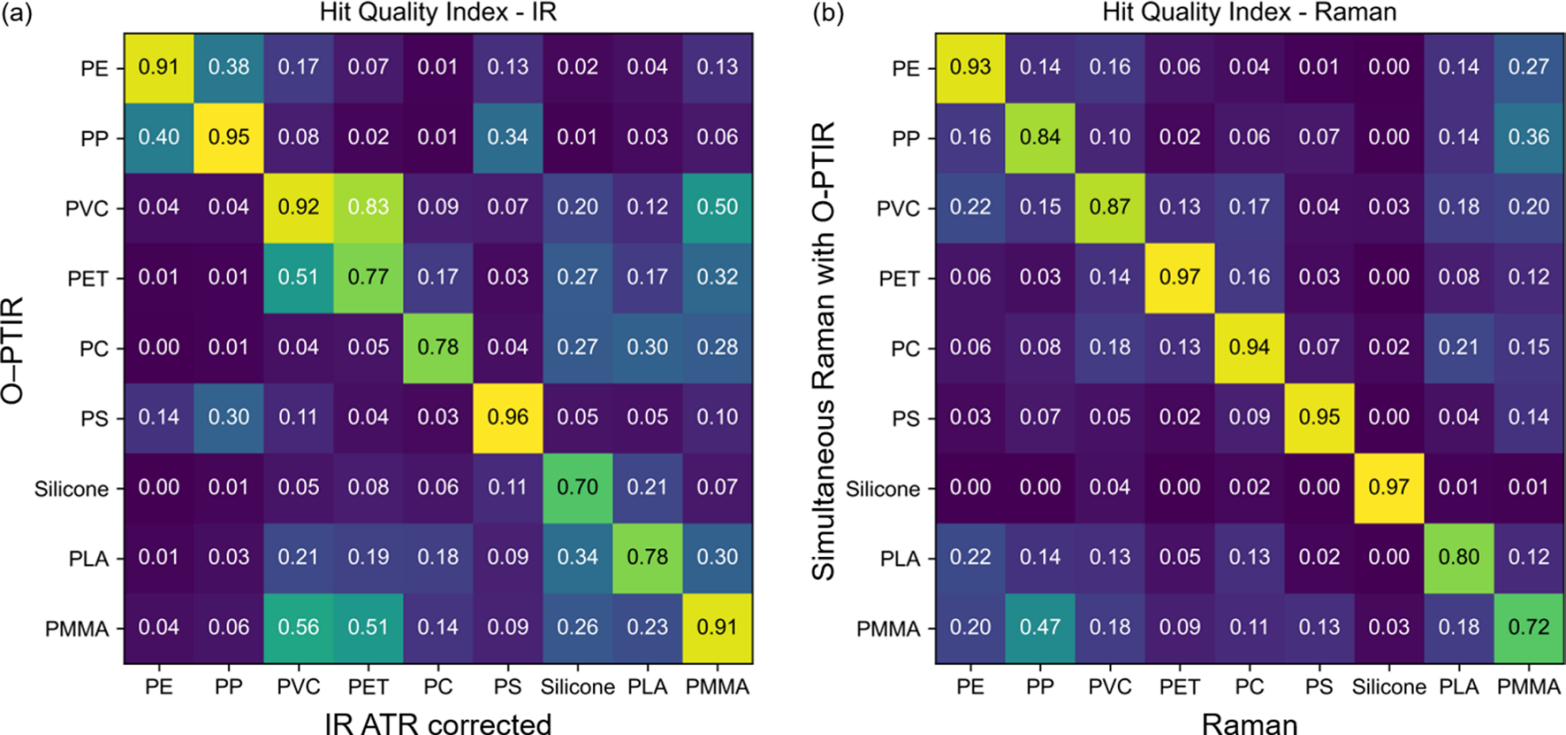
Hit quality index (HQI) of the acquired IR spectra from the FTIR system and the O-PTIR instrument (**a**) and the Raman spectra of the standalone Raman system and the Raman option of the O-PTIR instrument (**b**). The values are color-coded from dark violet to yellow to improve visibility.

Here, the HQI was used to quantify the agreement between the IR spectra acquired with the FTIR system after ATR correction and the IR spectra measured with the O-PTIR system and between Raman spectra of the standalone Raman instrument and the simultaneous Raman option of the O-PTIR instrument. The matrix in Fig. 3a summarizes the HQI values of the IR spectra in Fig. 1. The matrix in Fig. 3b shows the HQI values of the Raman spectra presented in Fig. 2. As pre-processing, an intensity normalization based on feature scaling was applied to the spectra. In addition, the spectra were interpolated using a linear interpolation method to match the wavenumber values. All spectra were compared to assess the suitability of the O-PTIR method for polymer identification. The diagonal line indicates the resemblance between matching polymer types.

Figure 3a summarizes the HQIs of the IR spectra. The values of the diagonal line range from 0.7 for silicone to 0.96 for PS, indicating sufficient to almost perfect matching. The highest off-diagonal HQI is for PVC to PET at 0.83, showing the highest chance for misidentification using the O-PTIR system. The presence of phthalates as a plasticizer in PVC and from ester bonds in PET showed a high spectral resemblance. However, even though the calculated HQI was high, the most significant hit for PVC was its FTIR counter-spectrum with an HQI of 0.92. While only five plastic IR spectra have an HQI above 0.5 with a false partner, the great majority of HQIs for IR spectra lie below 0.5 and even below 0.2.

The HQIs of the Raman spectra are summarized in Fig. 3b. The HQIs along the diagonal line range from 0.72 for PMMA to 0.97 for PET and silicone. Most of the remaining off-diagonal HQI values were lower than 0.2, and only one was close to 0.5, which ruled out the possibility of misidentification. Thus, the simultaneous Raman option of the O-PTIR instrument is in good agreement with the spectra of the standalone Raman instrument.

The HQI values of the standalone FTIR and Raman instruments are shown in supplementary Tables S1 und S2, respectively. The diagonal values are equal 1, and the values below and above the diagonal are identical for the spectra of plastic samples from the same instrument. The IR-HQIs tend to be larger in Fig. 3a than in Table S1, whereas the Raman-HQIs in Fig. 3b agree for most polymers well with Table S2. In agreement with Fig. 3a, relatively high off-diagnoal HQIs above 0.2 in Table S1 were found between IR spectra of PVC and PET, PVC and PMMA, PVC and PLA, and PET and PLA. In agreement with Fig. 3b, only one off-diagonal HQI in Table S2 was found to be above 0.2 for Raman spectra of PP and PMMA.

### Robustness of the HQI

**Figure 4:**
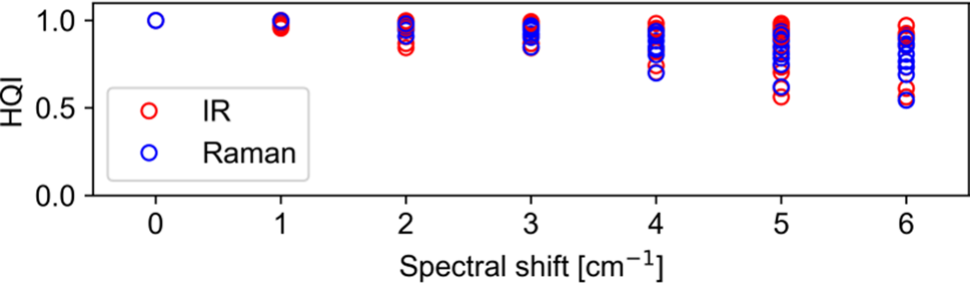
Robustness of HQI. The spectra are matching against themselves, shifted by the denoted wavenumbers (IR red, Raman blue symbols).

The HQI is mathematically based on the scalar or dot product of two spectra as explained in (Eq. 1). Following this train of thought, the HQIs are mainly influenced by the position of maxima rather than their intensity magnitude. In Fig. 4, the nine IR spectra acquired with the O-PTIR instrument for the same material are compared against themselves. Each dot represents the calculated HQI of the IR spectrum and Raman spectrum, respectively, against itself, shifted by the denominated wavenumber. Without a spectral shift, they obviously result in an HQI = 1.0. If one of the spectra is shifted in wavenumber compared to its original, their HQI reduces. Small spectral shifts up to 3 cm^−1^ still give HQI values well above 0.75. With a spectral shift up to 6 cm^−1^, HQI values are reduced for some materials down to 0.5. whereas other polymers still show HQI close to 1. These results are similar for IR and Raman spectra as evident from the overlap of the symbols.

### Two-dimensional identification

**Figure 5:**
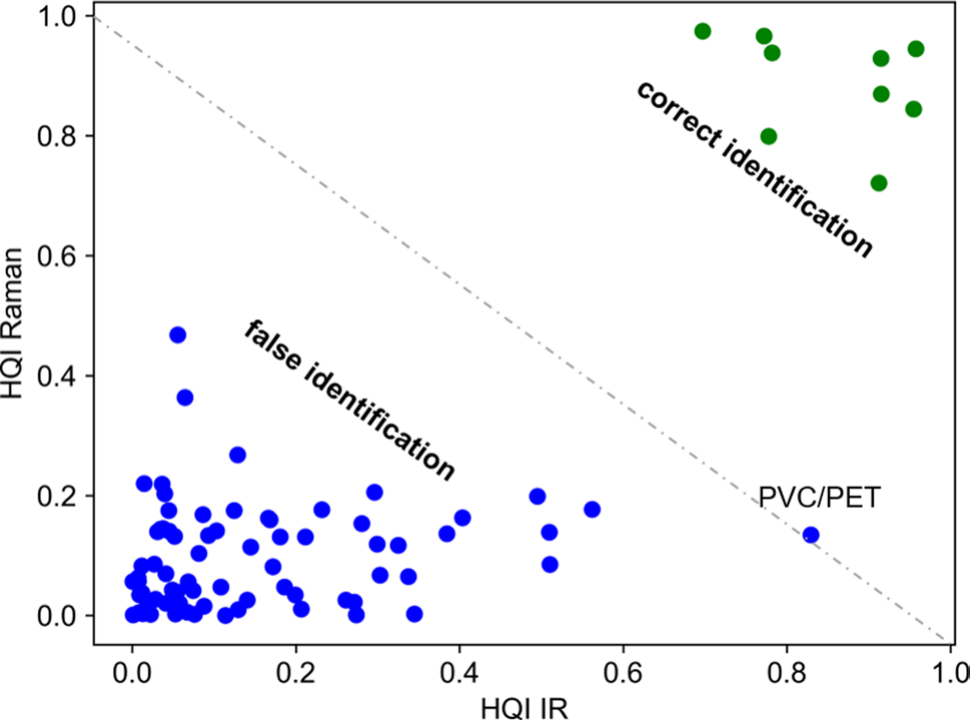
Two-dimensional identification plot for polymer material matching based on the hit quality index (HQI) of IR and Raman spectra. Illustration of the diagonal (green) and off-diagonal (blue) elements of Fig. 3.

In MP research, material identification is key to assessing the concentration and abundance of MP particles in a sample. Usually, MPs have been exposed to environmental influences that lead to degradation, building up a corona of contaminations and may alter the chemical polymer signature. Furthermore, spectral contributions of pigments and additives might overlap with the vibrational fingerprint of plastic. As a consequence, the vibrational signature of environmental particles often gives an ambiguous identification result. We propose a two-dimensional (2D) identification approach based on acquiring both IR and Raman spectra by the O-PTIR instrument to improve the correct identification rates. Thus, MP particles are identified based on their complementary chemical fingerprint, bringing higher certainty for library matching, as both the Raman and IR spectra can be considered. In the 2D identification approach, the HQI between the O-PTIR spectrum and the IR spectrum of the standalone FTIR instrument is plotted with the HQI between the Raman option of the O-PTIR instrument and the Raman spectrum of the standalone system resulting in a 2D-HQI graph. The spectra of the standalone systems are considered as reference spectra that can also be taken from spectral libraries or collected from the same instrument in an ideal case. Such a table with spectra from the same instrument is shown for the IR and Raman standalone systems in the supplementary Tables S1 und S2. The HQI values of Fig. 3 are illustrated in Fig. 5. The 2D-representation allows clearer differentiation between false material identification (blue) below the diagonal in the left bottom corner, and the correct matching (green) above the diagnonal in the top right corner. This is especially useful for materials with high HQI for multiple materials, e.g. as observed for PVC and PET based on IR spectra in Fig. 3a which is near the diagonal in Fig. 5. The 2D identification process ruled out particles with a high false HQI for one of the methods, here 0.83 for the IR spectra of PVC/PET, by low false HQI for the other method, here 0.13 for the Raman spectra of PVC/PET. The correct identification of PVC has an HQI(IR) = 0.92 and HQI(Raman) = 0.87.

### Application to microplastic particles

**Figure 6:**
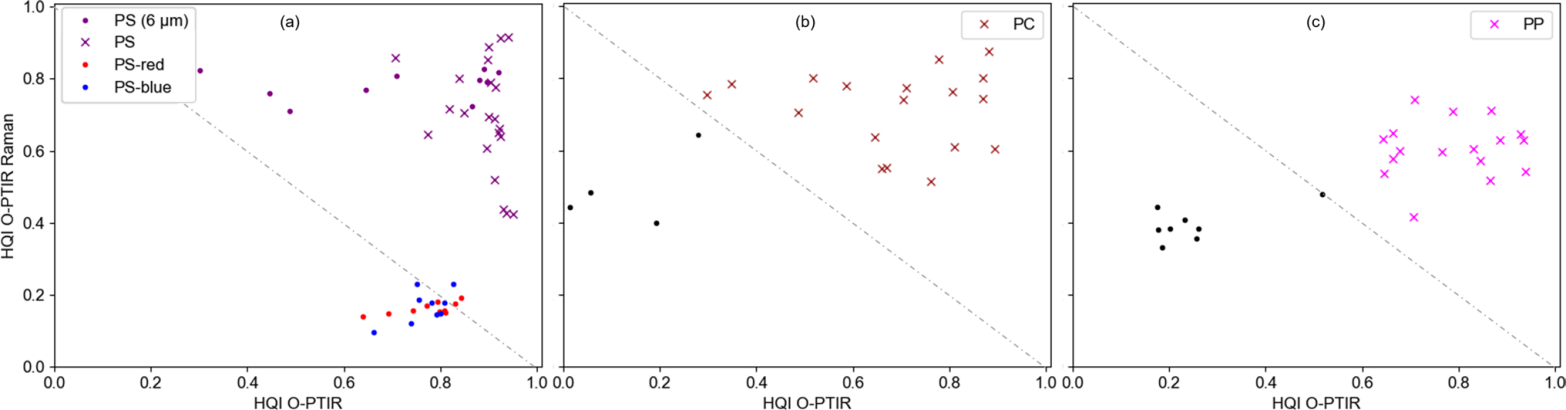
2D-HQI of different microplastic particles from simultaneous O-PTIR and Raman acquisition with reference spectra. HQI of transparent, irregular-shaped PS (x), transparent PS beads (6 µm) (o) and colored PS beads (10 µm) (o) (**a**); irregular-shaped PC (**b**); and irregular-shaped PP (**c**). Black dots: putative contaminations with both low O-PTIR and Raman HQI.

Different MP particles from commercial sources and MP particles prepared by wet grinding from bulk material were investigated as test samples for the 2D-HQI identification approach. Figure 6 shows the 2D-HQI graphs for different MP samples. The figure also shows that not all particles could be identified. This usually happens due to insufficient SNR or spectra of contaminations. In the figure, these spectra with HQI below the diagonal are marked as black dots. Most particles have a good HQI above 0.6, allowing IR and Raman spectra to match the reference. Although HQI values of each spectrum were calculated for all nine polymers, that were presented in Figs. 3 and 5, the 2D-HQI values in Fig. 6 were only plotted for the expected polymer for simplicity. Microscope images of the sample and the recorded spectra are compiled in the digital supplement (Figures S2–S4).

The simultaneously acquired Raman and IR spectra from self-made PS MPs match overall well (Fig. 6a) with the reference spectra acquired from bulk material (Fig. 1f, Fig. 2f). The Raman spectra of the commercial red and blue PS beads (both 10 µm diameter) are highly distorted through the interfering fluorescence and/or are dominated by resonance-enhanced bands of the pigments. However, combined with the O-PTIR signal, the materials can still be identified (Fig. 6a). The HQIs of smaller PS beads (6 µm diameter) shows the strength of the combined acquisition of IR and Raman spectra. Raman spectra are in good agreement with the reference, while the HQIs of some IR spectra are considerably lower.

For PC (Fig. 6b), HQI values of Raman spectra are spread between 0.6 and 0.9, and HQI values of IR spectra are between 0.3 and 0.9 due to lower SNR. For PP (Fig. 6c), the Raman spectra have lower SNRs, which also results in lower HQIs. Still, most spectra could be matched successfully with the PP reference using simultaneously acquired O-PTIR spectra of higher SNRs overcoming the shortcoming of using just one detection mechanism.

## Discussion

IR and Raman spectra acquired with the O-PTIR instrument were shown to be in good agreement with the standalone devices. In 2016 Käppler et al. asked if the MP analysis is performed best with FTIR or Raman spectroscopy. While they pointed out that a combined approach would lead to the highest accuracy, they concluded that the time and cost of dual-analysis make it less probable for routine analysis^11^. However, the technological advances of the O-PTIR instrument enable the simultaneous acquisition of IR and Raman spectra from the same location with the same submicron resolution. The need for reliable and fast assessment of polymer types in MP research^37,38^ may be answered by combining the proposed 2D identification approach using the O-PTIR principle.

The presented spectra demonstrate the complementary nature of Raman and IR spectroscopy. While some bands show strong changes in polarizability leading to high Raman activity, others have strong changes in dipole-moment corresponding to intense vibrational bands captured in IR spectra. According to the rule of mutual exclusion in molecular spectroscopy, the observation of molecular vibrations also relates to molecular symmetry. It states that normal modes cannot be both IR- and Raman-active in a molecule with a center of symmetry, and they are either IR- or Raman-active for such molecules. As the symmetry is broken due to coupling effects in polymers, most bands can be observed in IR and Raman spectra, however some of them with markedly different intensities.

A possible reason for spectral variations of some plastics is the probing of different sample positions due to the inter-system experiments. The O-PTIR microscope probes a small diffraction-limited spot in the submicrometer range, whereas FTIR spectroscopy collects an average signal from a larger area, particularly if the sample is in contact with an ATR interface. This harbors the risk that the O-PTIR was affected by point-to-point variations owing to its higher spatial resolution. This is less likely to be a concern for discrete homogeneous MPs than for larger, possibly heterogeneous samples. Heterogeneity effects can be overcome by collecting and averaging multiple spectra per particle, however at the expense of extending the total exposure time and reducing the throughput in high-content screening scenarios.

Further improvement in spectral matching and material identification of both IR-based approaches could be achieved by a polymer-based ATR correction with tailored refractive index compensation. In addition, Sobieski et al. presented a method for improved ATR correction, which accounts for artifacts introduced through spectral deviations and shifts for higher wavenumbers^39^.

Calculation of HQIs were preferred to other chemometric approaches such as principal component analysis (PCA)^40^ and cluster analysis^3^ because commercial spectral libraries with thousands of entries or in-house spectral libraries can be used and the HQI is widely established to quantitate the agreement of spectra. Other distance measures such Euclidean and Mahalanobis distances require more data processing including normalization. PCA is also based on an orthogonality test of the eigenvectors, however at higher dimensionality which is more difficult for visualization and quantitation than HQIs.

## Conclusion

It is still under debate which method is best suited to probe MP particles. IR and Raman-based approaches are attractive candidates due to their label-free fingerprint capability. Limitations include sensitivity, specificity, identification rate, reproducibility and throughput. Small particles below 20 µm are poorly resolved by direct IR sensing, and their IR spectra are affected by dispersive scattering artifacts and low SNR. Raman microspectroscopy of such small particles is able to give good SNR spectra without artefacts. Here, proper intensity and wavelength calibration of Raman instruments suppress instrument-dependent variations, which, if unaccounted for, reduce reproducibility and, consequently, the HQI value with the correct entry in the spectral library. The combination of both Raman and IR measurements can take full advantage of the well-known complementarity information of dipole moment and polarizability of molecular bonds. It enables more sensitive, more accurate and reproducible identification of particles, however, at the expense of lower throughput compared to a single-device approach due to consecutive collection of IR and Raman spectra using conventional separate state-of-the-art devices. Therefore, it can be concluded that simultaneous acquisition of IR and Raman spectra of MP particles with the O-PTIR instrument is a superior approach for material identification in terms of throughput than two standalone instruments .

Another advantage of the O-PTIR instrument utilizing the optical photothermal IR detection scheme is the submicron resolution capability and lack of Mie scattering effect. This has been demonstrated for O-PTIR spectra of MP particles down to 6 µm. Limits for MP detection by IR and Raman spectroscopy from various sources such as water, sediment, food and organisms are the quality of the sample which might be contaminated or insufficient SNR which might suffer from unspecific scattering and background. The resulting low HQI often do not allow an unambiguous classification. The added value of the 2D-approach is that either the IR or Raman spectrum is less affected by distortions for more accurate identification. This was shown for PVC. IR and Raman fingerprint information might also be complementary e.g. IR spectra identify the polymer type and Raman spectra identify the pigments which was evident in Fig. 6a for colored PS beads. A prerequisite is, of course, that the spectral library contains reference Raman spectra of pigments.

We plan to focus on the capability of O-PTIR to probe MP particles below 20 µm because direct IR approaches give spectra of poor SNR for such particles. A potential route of O-PTIR to improve throughput for particle screening is rapid scanning of extended areas of interest at single wavenumbers using intense QCL radiation followed by collecting full IR and Raman spectra from localized particles. Besides MP particles, the 2D analysis could also contribute to advances in other bio-analytical applications.

## Materials and methods

### Optical photothermal infrared (O-PTIR) spectrophotometer

**Figure 7:**
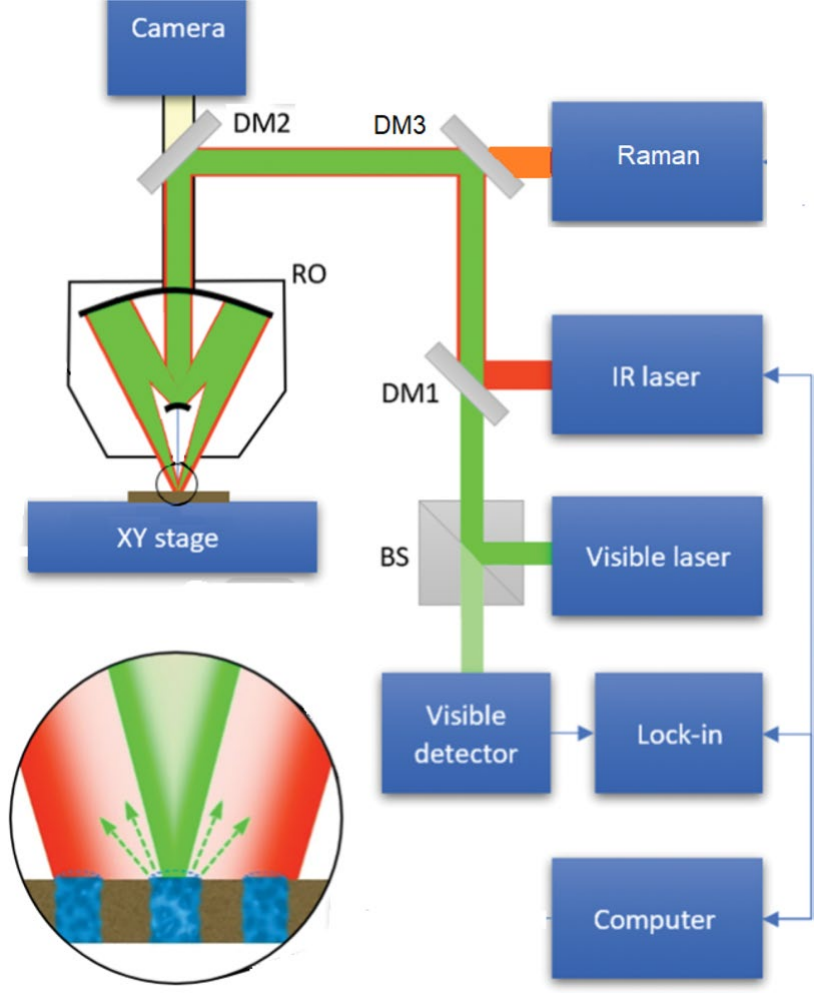
Schematic diagram of O-PTIR spectroscopy. A pulsed tunable IR laser is collinear with the visible detection laser, and the beams are focused via dichroic mirrors (DM1-3) on the sample surface through a reflective objective (RO). When IR absorption occurs, the photothermal response of the sample surface is monitored by the visible probe laser. The reflected light passes through a beam splitter (BS), is measured by a visible detector, and the IR signal is extracted while sweeping the wavelength of the IR laser source. Raman-shifted light is simultaneously diverted to the Raman spectrometer.

O-PTIR spectra and simultaneous Raman scattering presented in this work were collected with the mIRage + R™ Infrared and Raman microscope (Photothermal Spectroscopy Corp., Santa Barbara, CA, USA)^41^. The optical layout is shown in Fig. 7. The instrument is equipped with a four-chip, pulsed and broadly tunable high-power quantum cascade laser (QCL) covering 1800–800 cm^−1^ as the IR pump beam (MIRcat 2400, Daylight) and a 785 nm continuous wave (CW) probe laser. Data was collected by focusing the IR and probe laser on the sample using a 40 × /NA 0.78 reflective Cassegrain-style objective.

The system was purged with dry air to remove water vapor from the sample chamber and avoid interferences. Samples were placed on MirrIR reflective glass slides with a metal coating (Kevley, USA). Background spectra collected from MirrIR slides were used to account for the power spectrum of the QCLs. Two scans were co-added at 6 cm^−1^ resolution with a QCL scan rate of 1000 cm^−1^/s. Typical O-PTIR acquisitions settings for plastic samples are presented in supplementary figure S1. All sample O-PTIR spectra were automatically normalized to the collected background spectrum. Whereas the QCL power was carefully controlled and kept below 21% to avoid melting the polymer at a 10% duty cycle, Raman spectra could be collected with up to 100% power of the probe laser (ca. 15 mW at the sample). The Raman spectral range covered 237–1925 cm^−1^ using a grating of 600 lines per mm centered at 1150 cm^−1^ and 1024 × 256 pixel deep depletion, back-illuminated CCD detector. Two scans were co-added with activated cosmic ray correction.

The spectra from MP particles are collected using semi-automated spectra detection. Particle locations are pre-set, and the autofocus, based on the IR spectral response, is used to simultaneously acquire IR and Raman spectra.

### FTIR spectrophotometer and data pre-processing

IR spectra of larger plastic particles presented in this work were collected with the FTIR spectrometer Cary 670 (Agilent, USA) combined with an ATR accessory using a ZnSe triple reflection element (Miracle, Pike Technologies) and a DTGS detector. ATR-IR spectra were recorded from 600 to 4000 cm^−1^. The IR spectra of smaller plastic particles were acquired with FTIR microscope Cary 620 (Agilent, USA), liquid nitrogen cooled MCT detector, and a 15 × /NA 0.62 Cassegrain objective lens with a slide-on ATR germanium accessory. Here, the IR spectra ranged from 800 to 4000 cm^−1^.

Standard ATR correction was based on the change in refractive index at the ATR crystal-sample interface. The refractive index of typical polymers varies between 1.34 for fluorinated ethylene propylene to 1.65 for poly acryl sulfone^42^. Most refractive indices of the polymers presented distribute around n_s_ = 1.5, which has been assumed together with the known angle of incidence θ = 45° and the refractive index n_ATR_(ZnSe) = 2.4 and n_ATR_(Ge) = 4.0 near 10 µm for the standard correction according to

2 $$d=\frac{{\lambda }_{IR}}{2\pi {\left({n}_{ATR}^{2}{\mathrm{sin}}^{2}{\theta }_{ATR}-{n}_{S}^{2}\right)}^{1/2}}$$

**Figure 8:**
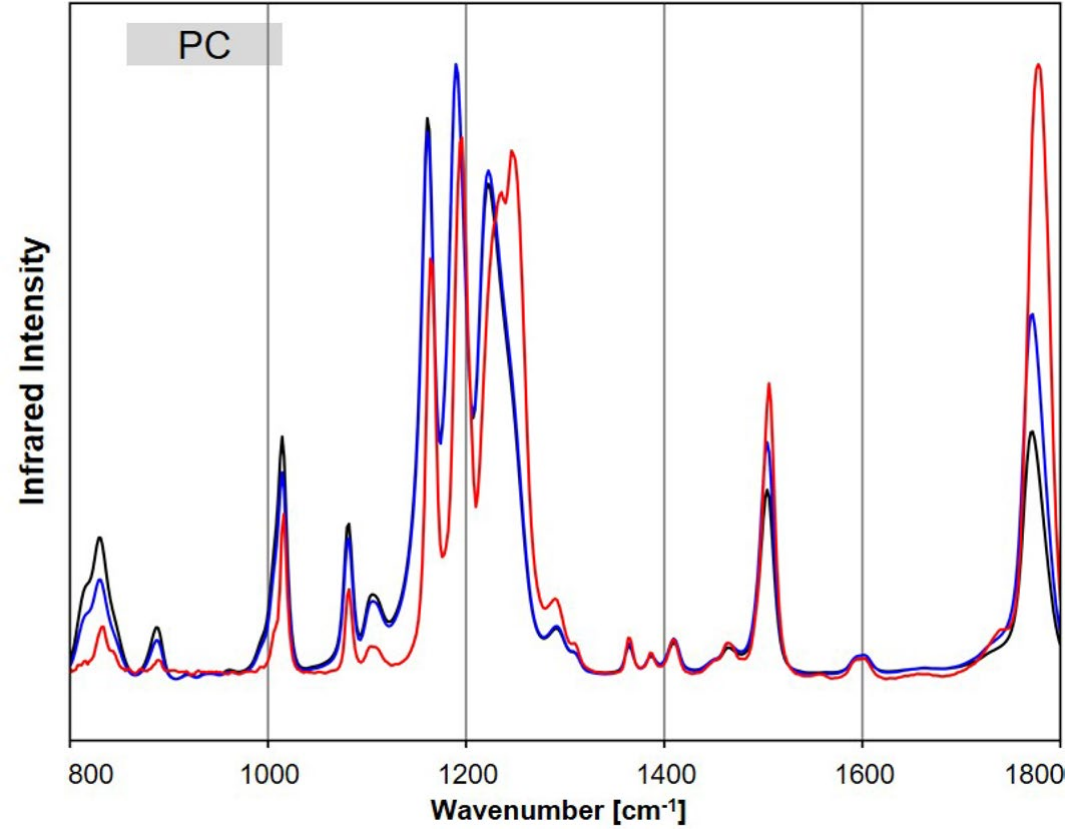
Exemplary spectra of PC, with the acquired raw FTIR spectrum (black), the ATR-corrected (blue) and the O-PTIR spectrum (red).

To illustrate the impact of the ATR correction, the IR spectra of PC were shown in Fig. 8 as an example. Before ATR correction (black), the lower wavenumber bands, e.g. near 830 and 1015 cm^−1^, were more intense, while the higher wavenumber bands, e.g. near 1503 and 1771 cm^−1^, were less intense relative to the reference band at 1190 cm^−1^. After ATR correction (blue), the lower wavenumber bands decreased, and the higher wavenumber increased relative to the band at 1190 cm^−1^. Consequently, the similarity with the O-PTIR spectrum (red) improved. The agreement between spectra was quantified using the HQI. However, higher wavenumber bands in the ATR-FTIR spectra, particularly for C=O bands above 1700 cm^−1^ (Figs. 8 and 1), are shifted towards lower wavenumbers. An advanced correction^39^ could compensate for these artifacts, which was not performed here.

### Raman spectrophotometer

Raman spectra were acquired with the Raman spectrometer RXN1 using a fiber-coupled 785 nm laser as the excitation source, combined with a microscope (Kaiser Optical System, USA). The laser output power of 150 mW was focused via a 10 × /NA 0.25 microscope objective on larger particles, and a 100 × /NA 0.90 objective lens (Nikon, Japan) was used for smaller ones. A holographic transmissive grating dispersed low and high wavenumbers over the CCD detector, which provided the spectral range from 250 to 3500 cm^−1^ at a spectral resolution of 4 cm^−1^ for each spectrum. The wavenumber axis was calibrated with a neon emission light source. The intensity axis was calibrated with a white light source of known emission profile. The laser excitation wavelength was calibrated with the intense cyclohexane band at 801.8 cm^−1^ as a reference, following the routines of the HoloGrams control software and the calibration accessory (Kaiser Optical System, USA). Raman spectra were presented from 250 to 1800 cm^−1^.

### Data pre-processing

**Figure 9:**
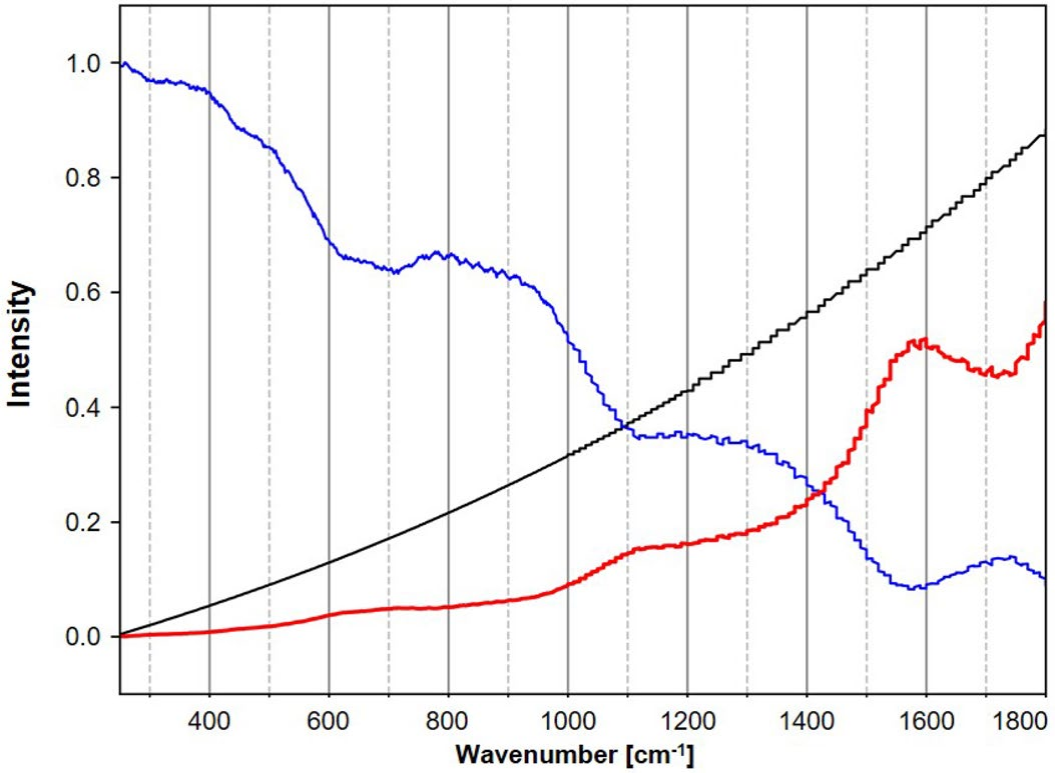
Reference spectrum of the white light source (black), the experimental spectrum of the white light source from the O-PTIR instrument (blue), and the calculated correction factor per wavenumber (red).

In Fig. 9, the reference spectrum of the white light source (black) is plotted together with the experimental spectrum, which was collected from the white light source using the O-PTIR Raman spectrometer (blue). After interpolating the reference data to the wavenumber axis of the experimental spectrum, a calibration factor was calculated and included in the plot for each wavenumber (red). Finally, the experimental Raman spectra of plastic were multiplied with these calibration values, baseline corrected and normalized. The more than five times lower collection efficiency between 1500 and 1800 cm^−1^ of the O-PTIR instrument was compensated through a larger calibration factor. In addition, the wavenumber axis was calibrated based on the spectrum of PS, which is suitable due to its distinctive narrow bands^33^. An additional baseline correction was applied to the Raman spectra acquired from MP particles using the asymmetrically reweighted penalized least squares (arPLS) method proposed by Baek et al.^43^. The Savitzky-Golay filter savgol_filter from the scipy signal processing library^44^ was applied for smoothing before calculating the HQI.

### Sample selection and preparation

The nonpolar polymer polyethylene (PE) is the most common plastic and is primarily used for packaging. The saturated hydrocarbon chains give the polymer similar chemical properties to paraffin. The second most widely produced plastic polypropylene (PP) consists of methyl groups along the carbon chain, which determines the shape and structure of the molecule. Its properties are similar to PE. Polyvinyl chloride (PVC) is the world's third most widely produced synthetic plastic polymer. It has an atactic stereochemistry with random distribution of the chloride centers, which mainly define the properties of the polymer. Polyethylene terephthalate (PET) is the most common thermoplastic polymer resin of the polyester family, which is the fourth-most-produced polymer mainly used in textiles and as containers for drinks and foods. It has a high intrinsic viscosity and is decomposed mainly into carbon, hydrogen, and oxygen during thermal disposal. Polycarbonates (PC) consist of carbonate bonds and aromatic rings. The carbonate esters have planar cores, which give the polymer rigidity. Another polymer with aromatic hydrocarbons is polystyrene (PS). Its properties are determined by van der Waals forces, which accumulate between the polymer chains. Standard PS is atactic with randomly oriented phenyl groups on both sides of the carbon string. Chains with an inorganic silicon-oxygen backbone and organic methyl groups attached to their center build silicone. Polylactic acid (PLA) has become a popular polymer and bioplastic because renewable resources economically produce it. The monomer is typically made from fermented plant starch such as corn. It contains ester groups on the carbon chain. Also, polymethylmethacrylate (PMMA) consists of methyl and ester groups linked to the main carbon chain.

For inter-system comparison homogenous bulk samples were chosen to ensure probing and spectra collection from the same sample. Still the location on the surface for spectral acquisition was not identical through the change of instrument and local variations could not be excluded.

Bulk polymer material for reference samples came from commercial sources and personal sampling. MP particles came from commercial and in-house preparation from bulk materials using a wet-abrasion method. Red PS beads with a nominal diameter of 10.47 ± 0.08 µm and blue PS beads with a nominal diameter of 10.33 ± 0.13 µm from microParticles GmbH (Berlin, Germany) and transparent PS beads with a nominal diameter of 6.6 ± 0.2 µm from BS-Partikel GmbH (Mainz, Germany) were used, diluted in particle-free water and dried on the substrate. In addition, PS, PC and PP were prepared using the in-house sample preparation method. The irregular-shaped particles had an approximated diameter from 5 to 50 µm. The polymer material was cross-checked with the material databases 'KnowItAll' (Wiley, NJ, USA).

## Supplementary Information

Supplementary Information.

## Data Availability

The datasets used and/or analysed during the current study available from the corresponding author on reasonable request.
