# Supplementary material for: Optical photothermal infrared spectroscopy with simultaneously acquired Raman spectroscopy for two-dimensional microplastic identification
Source: Sci Rep. 2022 Nov 5;12:18785. doi: 10.1038/s41598-022-23318-2 (PMC9637219; doi:10.1038/s41598-022-23318-2)
Supplement: Supplementary file 1 — Supplementary Information. [file 41598_2022_23318_MOESM1_ESM.docx]

**Supplementary Information**

**Optical Photothermal Infrared Spectroscopy with Simultaneously Acquired Raman Spectroscopy for Two-dimensional Microplastic Identification**

Julia Sophie Böke^1^, Jürgen Popp^1,2^, Christoph Krafft^1^

^1^Leibniz Institute of Photonic Technology, Jena, Germany

^2^Institute of Physical Chemistry and Abbe Center of Photonics, University Jena, Jena, Germany

1. **Data acquisition**

Typical meta-data of the data acquisition settings for O-PTIR of microplastics:


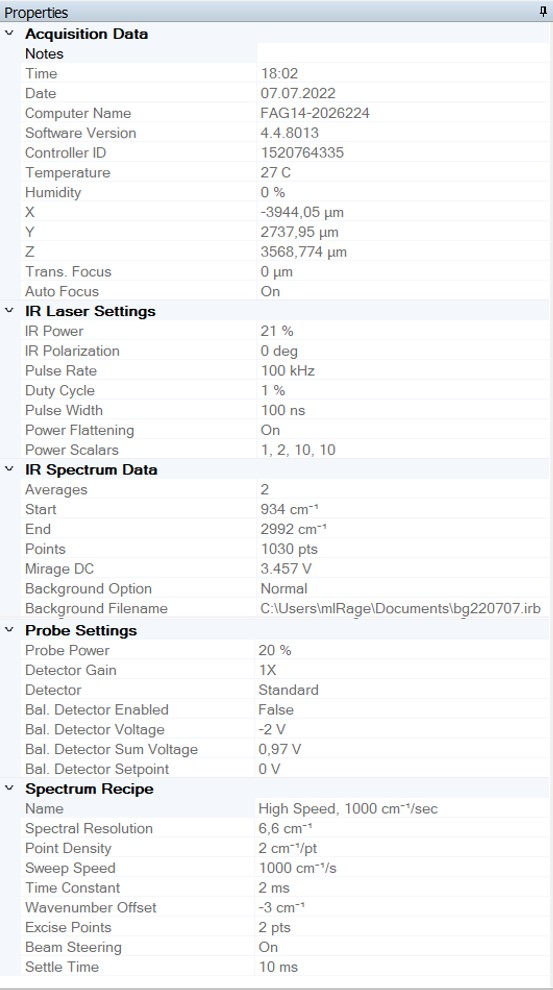


Figure S1: Meta-data of the acquisition settings.

1. **Microplastic samples and spectra**

Polystyrene (PS) particles made from wet-grinding of bulk materials (x) have been investigated together with commercial PS beads (•) that tend to form aggregates. They have been suspended in milliQ® water and dried on the reflective substrate. The microscopy images were acquired with the O-PTIR instrument and show the probing positions for spectra acquisition (Figure S2). O-PTIR spectra are plotted in red and Raman spectra are plotted in green.

**
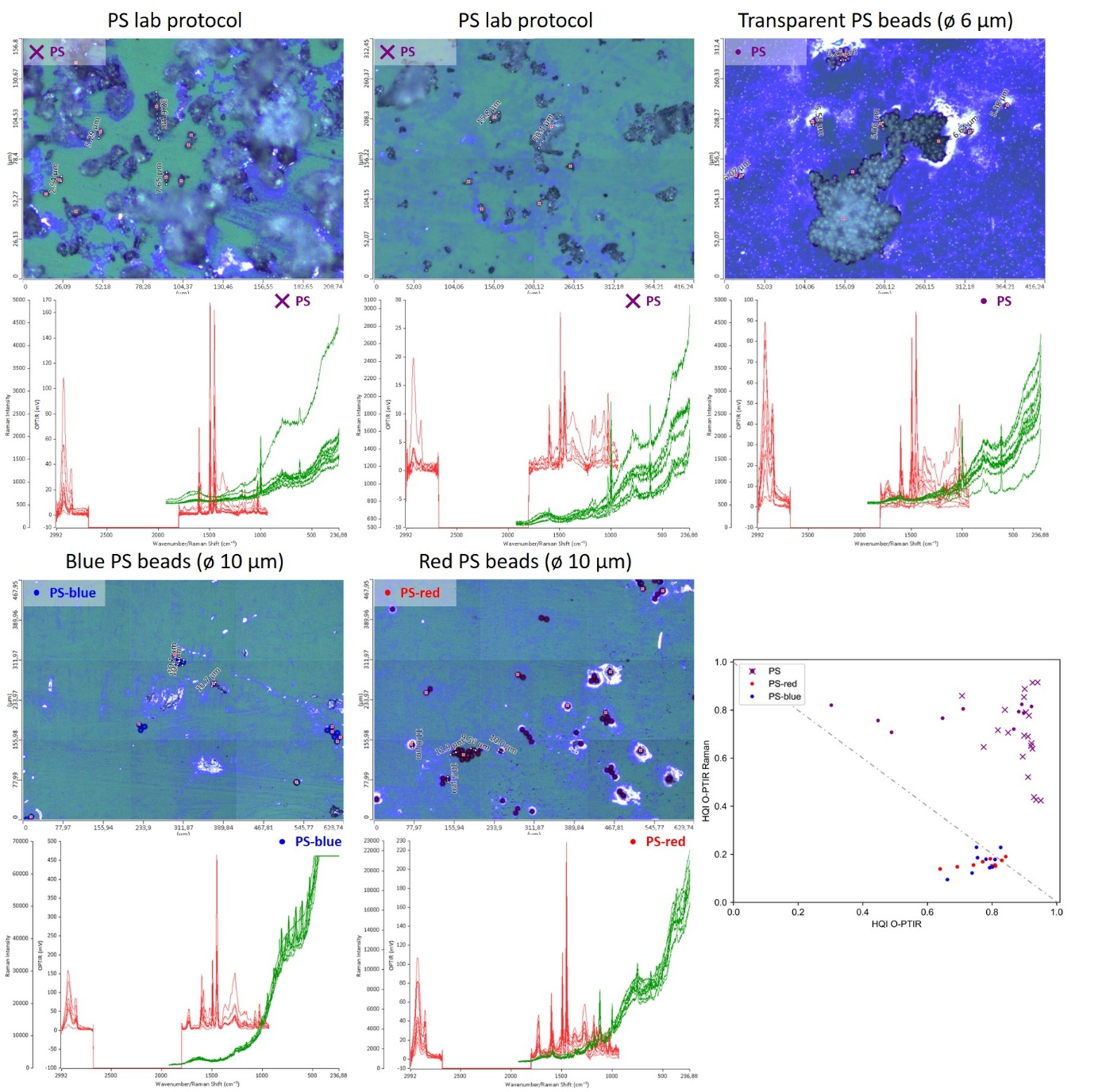
**

Figure S2: O-PTIR microscopy images of PS with measuring positions and size indicators are shown together with the simultaneously acquired O-PTIR (red) and Raman (green) spectra. The marker indicates their position in the resulting 2D-HQI graph.

**
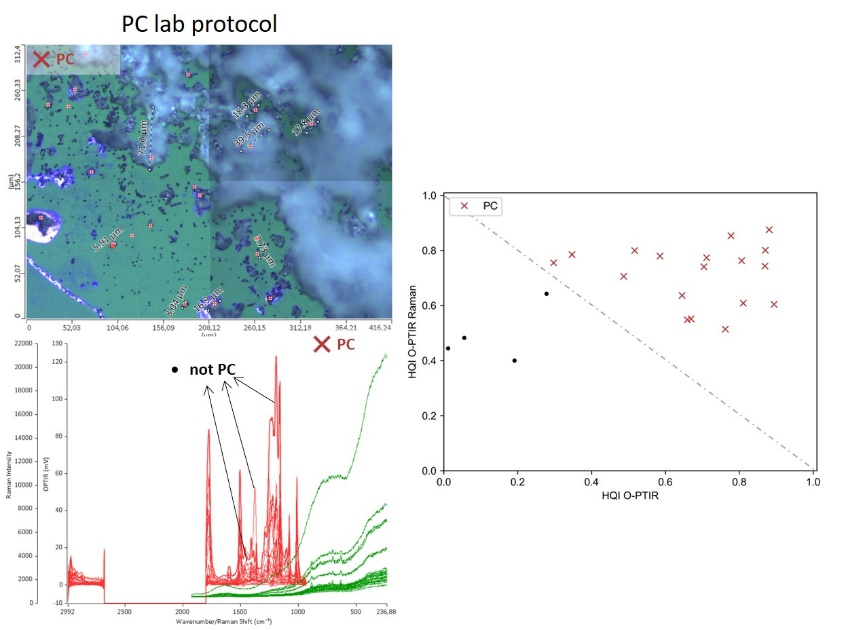
**

Figure S3: O-PTIR microscopy images of PC with measuring positions and size indicators are shown together with the simultaneously acquired O-PTIR (red) and Raman (green) spectra. The marker indicates their position in the resulting 2D-HQI graph.

The PC sample in Figure S3 and PP sample in Figure S4 show some contaminations from a different material, that are indicated in the 2D-HQI plot as black dots. They are also evident in the acquired spectra.

**
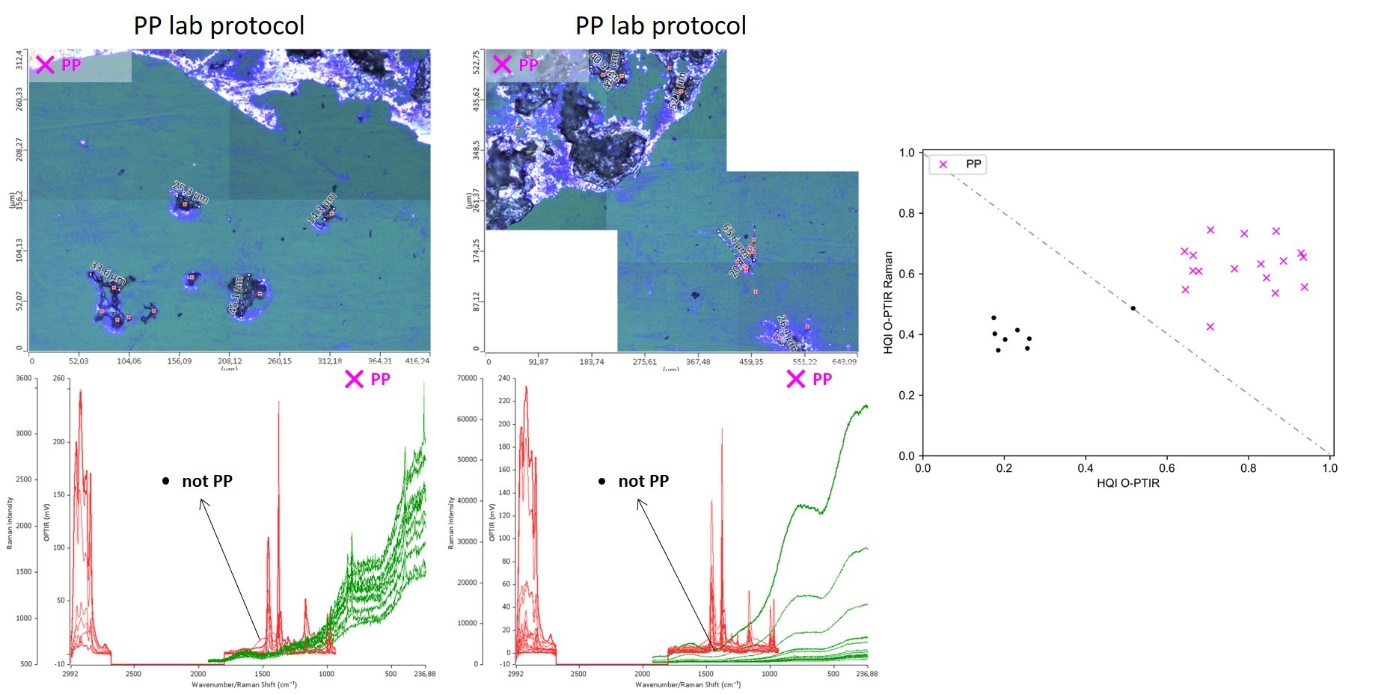
**

Figure S4: O-PTIR microscopy images of PP with measuring positions and size indicators are shown together with the simultaneously acquired O-PTIR (red) and Raman (green) spectra. The marker indicates their position in the resulting 2D-HQI graph.

**3. Hit quality index of polymers with standalone FTIR and Raman instruments**

Table S1: Hit quality index of FTIR spectra in the fingerprint range (950-1800 cm^-1^)

|  | PE | PP | PVC | PET | PC | PS | Silicone | PLA | PMMA |
| --- | --- | --- | --- | --- | --- | --- | --- | --- | --- |
| PE | 1 | 0.11 | 0.02 | 0.01 | 0.00 | 0.00 | 0.00 | 0.01 | 0.01 |
| PP | 0.11 | 1 | 0.05 | 0.02 | 0.00 | 0.01 | 0.02 | 0.04 | 0.02 |
| PVC | 0.02 | 0.05 | 1 | 0.64 | 0.02 | 0.01 | 0.11 | 0.21 | 0.21 |
| PET | 0.01 | 0.02 | 0.64 | 1 | 0.03 | 0.00 | 0.15 | 0.24 | 0.15 |
| PC | 0.00 | 0.00 | 0.02 | 0.03 | 1 | 0.00 | 0.03 | 0.12 | 0.03 |
| PS | 0.00 | 0.01 | 0.01 | 0.00 | 0.00 | 1 | 0.04 | 0.01 | 0.00 |
| Silicone | 0.00 | 0.02 | 0.11 | 0.15 | 0.03 | 0.04 | 1 | 0.18 | 0.03 |
| PLA | 0.01 | 0.04 | 0.21 | 0.24 | 0.12 | 0.01 | 0.18 | 1 | 0.09 |
| PMMA | 0.01 | 0.02 | 0.21 | 0.15 | 0.03 | 0.00 | 0.03 | 0.09 | 1 |

Table S2: Hit quality index of Raman spectra in the fingerprint range (250-1800 cm^-1^)

|  | PE | PP | PVC | PET | PC | PS | Silicone | PLA | PMMA |
| --- | --- | --- | --- | --- | --- | --- | --- | --- | --- |
| PE | 1 | 0.14 | 0.18 | 0.04 | 0.03 | 0.01 | 0.00 | 0.18 | 0.16 |
| PP | 0.14 | 1 | 0.12 | 0.02 | 0.06 | 0.07 | 0.00 | 0.13 | 0.46 |
| PVC | 0.18 | 0.12 | 1 | 0.13 | 0.13 | 0.04 | 0.03 | 0.13 | 0.20 |
| PET | 0.04 | 0.02 | 0.13 | 1 | 0.12 | 0.02 | 0.00 | 0.05 | 0.08 |
| PC | 0.03 | 0.06 | 0.13 | 0.12 | 1 | 0.06 | 0.02 | 0.11 | 0.10 |
| PS | 0.01 | 0.07 | 0.04 | 0.02 | 0.06 | 1 | 0.00 | 0.02 | 0.13 |
| Silicone | 0.00 | 0.00 | 0.03 | 0.00 | 0.02 | 0.00 | 1 | 0.00 | 0.02 |
| PLA | 0.18 | 0.13 | 0.13 | 0.05 | 0.11 | 0.02 | 0.00 | 1 | 0.12 |
| PMMA | 0.16 | 0.46 | 0.20 | 0.08 | 0.10 | 0.13 | 0.02 | 0.12 | 1 |
